# Reducing crash risk for young drivers: Protocol for a pragmatic randomised controlled trial to improve young driver sleep

**DOI:** 10.1016/j.heliyon.2024.e27066

**Published:** 2024-02-24

**Authors:** Simon S. Smith, Kalina R. Rossa, Shamsi Shekari Soleimanloo, Cassandra L. Pattinson, Dwayne L. Mann, Shannon L. Edmed, Paul M. Salmon, Karen A. Sullivan

**Affiliations:** a Institute for Social Science Research, Faculty of Humanities, Arts and Social Sciences, The University of Queensland, Brisbane, Australia; b ARC Centre of Excellence for Children and Families over the Life Course, The University of Queensland, Brisbane, Australia; c ARC Centre of Excellence for the Digital Child, The University of Queensland, Brisbane, Australia; d Centre for Human Factors and Sociotechnical Systems, The University of the Sunshine Coast, Sunshine Coast, Australia; e School of Psychology and Counselling, Faculty of Health, Queensland University ofTechnology, Brisbane, Australia

**Keywords:** Risky driving, Sleepy driver, Young driver, Road crash risk, Sleep banking, Sleep extension, Intervention, Accelerometry, Actigraphy

## Abstract

**Background:**

Road trauma is a leading cause of death and disability for young Australians (15–24 years). Young adults are overrepresented in crashes due to sleepiness, with two-thirds of their fatal crashes attributed to sleepy driving. This trial aims to examine the effectiveness of a sleep extension and education program for improved road safety in young adults.

**Methods:**

Young adults aged 18–24 years (n = 210) will be recruited for a pragmatic randomised controlled trial employing a placebo-controlled, parallel-groups design. The intervention group will undergo sleep extension and receive education on sleep, whereas the placebo control group will be provided with information about diet and nutrition. The primary outcomes of habitual sleep and on-road driving performance will be assessed via actigraphy and in-vehicle accelerometery. A range of secondary outcomes including driving behaviours (driving simulator), sleep (diaries and questionnaire) and socio-emotional measures will be assessed.

**Discussion:**

Sleep is a modifiable factor that may reduce the risk of sleepiness-related crashes. Modifying sleep behaviour could potentially help to reduce the risk of young driver sleepiness-related crashes. This randomised control trial will objectively assess the efficacy of implementing sleep behaviour manipulation and education on reducing crash risk in young adult drivers.

## 1 Introduction

Globally, more deaths of young adults (aged 15–24 years) are caused by road crashes than are caused by disease, drug use, suicide, violence, and war [1]. In Australia, young adults constitute approximately 12% of the population [2], and account for nearly 19% of all crash-related fatalities [3]. While the causes of road crashes are multifactorial, sleepiness is a leading determinant, contributing to 20–30% of all motor vehicle crashes globally [[4], [5], [6]]. Young adults are over-represented in sleepiness-related crashes, accounting for over two-thirds of all crashes [[7], [8], [9]]. Crashes attributed to sleepiness are typically more severe or fatal than are other types of crash because drowsiness, or even sleep onset (e.g., microsleeps), prevents drivers from braking or attempting any correction before the collision [10]. Current road safety strategies fail to adequately address the contribution of sleep loss to road deaths.

Young adults frequently drive when they are sleepy, which increases their crash risk. In one study, more than 23% of all young adults drove when they ‘were close to falling asleep’ [11], with 1 in 4 reporting a near-crash or crash in the past year due to sleepiness [12]. A range of lifestyle factors unique to this developmental period, including educational demands combined with part-time employment, and increased socializing at night are some of the most common drivers contributing to lost sleep and fatigued driving in this group [13].

Sleepiness-related crashes occur primarily due to inadequate sleep [10,[14], [15], [16], [17]] and young adults are chronically sleep deprived. The 2016 American Academy of Sleep Medicine (AASM) [18], recommends 7–9 h s of sleep for most 18–25-year-olds. Despite this, up to 60% of young adults (17–24 years) rate themselves as poor sleepers, 25% report getting less than 6.5 h s of sleep each night, and 20% report not sleeping at all at least one night in the past month [19]. The cognitive deficits associated with chronic daily sleep loss are cumulative and dose-dependent [20], which means that young adults who obtain 30 min less sleep per night than the recommended amount can find themselves with a 3½ hr sleep debt by the end of the week [[21], [22], [23], [24]]. Sleeping between 6 and 7 h in a 24-h period can *double* an individual's risk of a road crash, and sleep being restricted to less than 6 h per night nearly quadruples the risk [16,17]. Young adults may also be uniquely vulnerable to the detriments of sleep loss, since the brain areas responsible for complex higher order cognitions including decision making, set-shifting and risk and reward perception are still developing [25]. Despite the over representation of young adults in road crash fatalities, current road safety strategies such as education, detection and enforcement have failed to directly target driver sleepiness as a cause of young driver fatalities.

It is possible that an intervention targeted at increasing young drivers' habitual sleep duration (i.e. sleep *extension*) may result in reduced sleepiness and reduced associated crash risk for young drivers, and a reduction in secondary risk-related behaviours. Sleep extension involves increasing *habitual sleep duration* to reduce the accumulation of sleep debt by increasing opportunities for sleep (i.e. prescribing increased ‘time in bed’ by advancing bed-time), improving behavioural factors that promote sleep (e.g. regularity of bedtimes/wake-up times), and reducing stimulants impairing sleep (e.g. caffeine, evening artificial light exposure, digital device use etc.). Controlled experimental studies have demonstrated the benefits of increasing sleep duration on resilience to future fatigue, and improved attention [[26], [27], [28]], both key protective factors in road crashes [29,30]. Recent evidence from ‘at-home’ sleep extension intervention studies (with 'prescribed’ additional time in bed at home, as opposed to the controlled laboratory setting) suggest that sleep prescription may extend sleep duration by up to 60 min [31,32], 90 min [[33], [34], [35]], or by up to 20% of the participants' habitual sleep duration per evening [36]. Likewise, gradual sleep extension in adolescents' home environment can result in increased sleep duration and time in bed, improved cognitive performance and divided attention [37]. These data suggest increasing time in bed may increase sleep duration in the home environment. The results of such an increase and subsequent effects on behaviours such as driving have not yet been determined. As such, we propose a pragmatic randomised controlled trial of an intervention targeted at young sleepy drivers to increase habitual sleep duration to improve resilience to sleepiness and reduce their on-road crash risk.

### 1.1 Methods/design

#### 1.1.1 Trial objectives

The primary trial objective is to test the effectiveness of a sleep extension and education intervention on objective indices of sleep and risky driving performance of chronically sleep-deprived young adults (18–24 years old).

The secondary objective of the trial is to assess the effects of the sleep extension and education intervention on daily subjective indices of sleep, and other risk factors for adverse driving outcomes (e.g., higher order cognitive functions, alcohol use, stress, and mood).

We hypothesise that, in comparison to the placebo control group, the intervention group will;

(1) increase their habitual sleep duration by at least a clinically meaningful 25 min per night on average,
(2) decrease their frequency of acute sleep loss episodes (<6 h s of sleep) across a typical week,
(3) demonstrate reductions in high risk driving performance on the road and in the driving simulator.
(4) improved performance on computerised executive-function tasks associated with behavioural self-regulation.

#### 1.1.2 Sample size

A minimum sample size of 189 participants was initially calculated. We will aim to recruit 210 participants, allowing for ∼10% attrition at follow-up (consistent with drop-out from our previous studies in this age cohort). Based on the authors’ previous studies using the same measurement approach (h^2^_p_ = 0.12–0.37) [38], this sample size will provide 90% power, with a significance level of *p* = 0.05, to detect a 'small’ change in the primary driving outcome based on a parallel groups design with 0.3 correlation among the repeated measures. In addition, this sample size will provide 90% power to detect a 'small-medium effect size’ of a 5% increase in mean objective sleep duration (primary sleep outcome - equivalent to approximately 25 min additional sleep per night). This is a clinically and physiologically significant change [39], representing almost three extra hours of sleep each week. Based on our previous laboratory work [38] the study is also sufficiently powered for all proposed secondary outcomes.

#### 1.1.3 Participants and recruitment

We will recruit young adult (18–24 years), males and females who currently hold an Australian driver's license (Provisional 1 and 2, or Open/Full). The inclusion and exclusion criteria are presented in Table 1. Inclusion criteria were defined to select participants who may be at increased risk of sleepiness-related crashes, consistent with the AASM sleep duration recommendations. Exclusion criteria were defined to minimise the impact of uncontrollable variables by randomisation (see Table 1).

**Table 1:** Study inclusion and exclusion criteria.

*Inclusion* Criteria	*Exclusion* Criteria
• Self-reported sleep of < 7 h s per night • Self-reported sleep of < 6 h s on at least two nights per week. • Regular drivers (driving on > 3 days per week) • Everyday access to a passenger vehicle and a mobile device (smartphone) • Willing to make changes to either a sleep or nutrition related lifestyle behaviour	• Overnight shift worker (i.e., any shifts falling between 12am and 6am) • Travelled across >3 time zones within the past 3 months or intention to travel within the first 3 months of the study • Medically diagnosed clinical sleep or eating disorder. • Use of over-the-counter substances with psychoactive properties (e.g., Ginko Biloba, St. John's Wort) • Use of medically prescribed stimulants, antidepressants, antianxiety, antipsychotic, mood stabilising medications, sleep medications or appetite suppressants • Pregnant or planning a pregnancy in the following 10-month period • Sole carer of a child • Regular smoker – due to increased risk of sleep-related issues and motor vehicle accidents.

Recruitment for the trial will be promoted across metropolitan Brisbane and the Sunshine Coast (Queensland, Australia), using various strategies specifically targeting young adult populations including: in-community flyer distribution around universities, shopping centres, and community noticeboards, as well as targeted social media advertising, through Instagram and Facebook community groups for young adults (e.g. University media and Instagram pages), and snowballing. Participants will be compensated for their time taken to complete the trial, receiving a total of $450 AUD, paid in two instalments; $250 paid upon completion of the intervention laboratory session and $200 paid upon completion of the follow-up laboratory session.

##### 1.1.3.1 Randomisation and blinding

Following successful completion of screening and informed consent procedures, participants will be randomly allocated to either the Sleep Intervention group or the Nutrition ‘Placebo’ group by a data manager blind to participant recruitment using a computer-generated randomisation schedule. Participants will not be informed of their group allocation before completion of their baseline laboratory assessments to ensure a stable and equal baseline across participants.

The nature of the intervention does not permit fully blinding participants to their allocation status. Therefore, the trial will be partially blinded to prevent performance and attrition biases. However, the study aims and outcomes will be concealed, and the initial recruitment premise will be '*a study of health, activity, and transport’* to reduce expectancies. Additionally, evaluation of the outcomes will be conducted by staff blinded to participant allocation (e.g., an independent statistician), and the use of objective outcome measures will minimise the risk of ascertainment bias. A full explanation of both the real aims of the research and reasons for the concealment will subsequently be made available to participants upon completion of their participation or upon withdrawal from the study.

##### 1.1.3.2 Trial design and settings

**Fig. 1:**
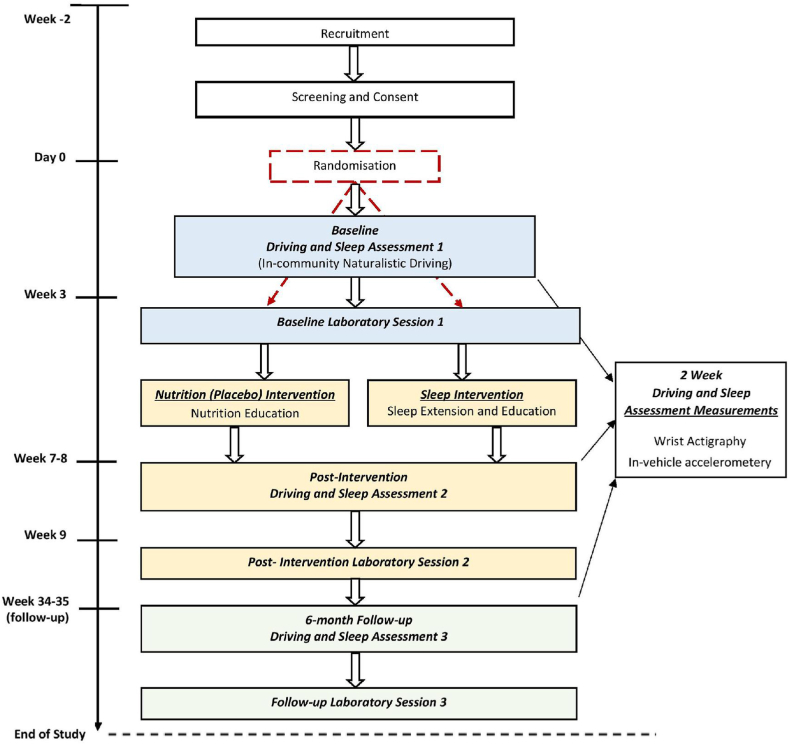
Flow diagram of recruitment, allocation, and assessment points at Baseline, Intervention, and Follow-up. Continuous naturalistic measurement refers to measurement undertaken continuously during day-to-day 'life as usual’ activities.

This trial employs a randomized, placebo-controlled, parallel groups design (see Fig. 1 for study design schematic). Across an approximate timeline of nine-months, participants will undergo three assessment periods (Baseline, Intervention/Maintenance, Follow-up), three laboratory sessions and a randomised sleep extension (intervention) or nutrition information (placebo control) component. The assessment periods will include naturalistic monitoring of participants’ sleep and driving performance (i.e. when driving their own vehicle in the community). Each assessment period will be followed by a session in the driving simulator laboratory at the study setting.

Upon recruitment and following informed consent procedures, participants will complete online pre-baseline questionnaires capturing demographic, health, sleep, risk and driving related information. Participants will then be provided with an actigraph (to be worn on their non-dominant wrist), and an in-vehicle accelerometer. The accelerometer will be installed into the boot/trunk of participants' vehicles. The actigraph will record habitual sleep and the accelerometer will record driving behaviour during a 2-week assessment period. During each assessment period participants will be instructed to continue their lives ‘as per usual’ while completing a daily sleep and/or food craving diary, continuously wearing their actigraph, and driving naturalistically in the community.

The three laboratory sessions (Fig. 1) will follow each of the baseline, maintenance, and follow-up assessment periods. All laboratory sessions will be scheduled within 7–14 days of the end of each data collection period. Participants will be advised to refrain from consuming alcohol on the night before, and any caffeinated beverages at least 4 h before attending the laboratory session. The laboratory sessions include a large array of objective and subjective measures to assess a range of secondary objectives (cognitive and socio-emotional outcomes). The measures used in this session have been carefully selected using validated instruments (i.e. NIH toolbox, PROMIS scales), and physiological assessments (EEG, ECG, eye tracking), alongside driving simulator scenarios developed specifically to elicit data on dimensions of vehicle control.

## 2 Trial intervention group: sleep extension and education

The aim of the intervention is to increase habitual sleep duration. Following the baseline assessment period, participants randomised into the sleep condition will be provided an individualised sleep extension and education session with an experienced sleep researcher. The sleep extension and education sessions consist of three core elements:

(1) Sleep scheduling: This component is based on behavioural principles for sleep scheduling [40]. The researcher will use sleep data collected from actigraphy during the participants' baseline assessment to calculate the participants' average bedtime, wake-up time and habitual nightly sleep duration. Based on these calculated estimates, participant will be 'prescribed’ an extra 20% of time to be spent in bed at home each night across the 3-week intervention period. To facilitate uptake of the intervention into existing sleep/lifestyle routines, participants will be asked to go to bed 15 min earlier each night gradually until their bedtime matches the additional 20% prescribed (this transition may take anywhere between 3 and 7 days). Participants will be asked to maintain this new 'time-in-bed’ for two more weeks. The total intervention period therefore spans 3 weeks (1 week of transitioning to the new sleep schedule, and 2 weeks of maintaining the new schedule). For example, a participant with an average bedtime of 11:30pm, and an average habitual nightly sleep duration of 6.5 h will be prescribed an additional 1.3 h ‘in-bed’ time each night, to be achieved gradually by going to bed 15 min earlier each night until it matches 10:10pm.
(2) Sleep Education Online modules: Participants will view 4 × 10-min educational video vignettes (purpose-developed by research team) providing basic education about sleep and sleep health. The topics covered include a) The fundamentals of sleep & circadian functions; b) The benefits of sleep for physical performance, road safety, cognitive and physical functioning; c) The benefits of good sleep for mental health & social functioning and d) The 'SIMPLE’ strategies; Practical tips and tools for supporting healthy sleep. These tips and tools were purpose-developed by the research team and centred on healthy sleep hygiene principles. The SIMPLE strategies include information about sleep 'Scheduling’ and regularity, 'Implementing a pre-bed routine’, 'Minimising screen time’ in the hours before bed, making lifestyle changes aimed at 'Prioritising sleep’, 'Learning to manage stress', and understanding how to make your 'Environment’ conducive to sleep. Participants view the video vignettes at their initial sleep scheduling session and are provided links to the videos via reminder text messages at two other time points in the intervention period (end of Week 1, and 3) encouraging them to revisit this content in their own time.
(3) Motivational interviewing and sleep pledge: After viewing the online modules, and receiving their new sleep schedule, the researcher will use motivational interviewing techniques to explore participant's self-efficacy, self-reported sleep needs and/or self-reported barriers to managing sleep health and timing. Using the framework of the 'SIMPLE’ strategies for improving sleep, participants will be provided with their new sleep Schedule, and asked to reflect on which two of the remaining 5 'SIMPLE’ strategies they would be able to commit to and implement for the duration of the intervention period. This process will facilitate autonomy and self-efficacy around sleep scheduling and timing, encouraging maximum fidelity to the intervention sleep schedule. To facilitate participant retention and commitment to the intervention schedule, participants will be asked to sign a 'pledge’ of their commitment to their agreed upon sleep schedule and choice of sleep improvement strategies for the duration of the intervention.

### 2.1 Placebo group: diet and nutrition education

The diet and nutrition education was chosen as a comparator (placebo arm) for this trial since, to the best of the authors’ knowledge, there are no current studies suggesting that providing participants with information about diet and nutrition has any direct effect on sleep duration, or on young adults’ driving behaviour. This program will be identical to the active intervention with respect to exposure (time-matched) and format (4 purpose built educational video vignettes), but the sleep-related information will be replaced with diet and nutrition related content. For example, participants in the diet and nutrition group engaged with will be required to engage with 4 × 10 min 'Nutrition Facts' online education modules delivering content covering, a) the fundamentals of diet and nutrition b) the benefits of a healthy diet for performance; c) the benefits (and consequences) of healthy/poor diet and nutrition and d) PIECES: Strategies for healthy eating (Plan, Include fruit and vegetables, Eat with other people; not the TV or that assignment, Choose smaller portions, Experiment, Skip sugary drinks), practical tips for maintaining a healthy diet. No pledge of behaviour change will be signed by the Placebo group. Furthermore, participants in the diet and nutrition education group will not receive education or instruction specifically about their sleep, nor any sleep-related feedback during the intervention phase.

#### 2.1.1 Outcome measures

Objective driving and sleep measures will be used in the trial to directly address the primary and initial secondary research objectives.

### 2.2 Primary outcome 1: risky driving performance

On-road risky driving performance will be assessed using small, sensitive, 3-axis accelerometers (GeneActiv Original, ActivInsights). The accelerometer is placed in a zip lock bag on a flat surface in the boot/trunk of the participant's vehicle and secured with duct tape. The device is oriented so that: x captures forwards-backwards acceleration, y captures left-right acceleration, and z captures up-down acceleration. These devices objectively record vehicular forces of acceleration from discrete vehicle events in forward and lateral directions, e.g., overcorrection after attentional drifts when drowsy [43] and provide an ecologically meaningful assessment of driving performance [44]. Previous research suggests that elevated g-force events have high predictive accuracy for crash and near-future crashes in young adults [43], and therefore provide a proxy measure of risky driving in young drivers [45].

Risky driving indices (elevated g-force events) extracted from these devices for analysis will include;

− Rapid starts (throttle acceleration): defined as forward acceleration (m/s^2^),
− Hard stops: defined as longitudinal acceleration due to braking (m/s^2^),
− Hard left turns: defined as lateral acceleration to the left side (m/s^2^), and
− Hard right turns: defined as lateral acceleration to the right side (m/s^2^).

Extending on prior research [43], a custom composite ‘risky driving variable’ will be created from these g-force indices and taking into consideration each participant's driving data per day and aggregated across the week, and at times of increased driving risk (e.g., driving at night/in the morning and at the circadian nadir). Changes in the risky driving composite from baseline to post-intervention will be used in primary analyses.

To minimise the risk of use by other drivers, participants will 1) be included in the study if they own their own private vehicle, 2) be asked to refrain from lending their vehicle to others during the study periods, and 3) complete a driving diary. Further, validation between wrist and car actigraphy is planned in secondary analyses of the study.

### 2.3 Initial secondary outcome: objective sleep

Objective assessment of sleep-wake states will be determined using wrist-worn accelerometers (i.e. actigraphs). Using specific scoring algorithms, actigraphy can provide a non-invasive and objective method of measuring habitual sleep and activity levels in naturalistic settings [41], and is comparable to polysomnography as a valid and reliable measure of sleep-wake parameters, particularly for sleep onset and wake times [42]. The GeneActiv Original actigraphs are wrist-watch style accelerometers that record raw triaxial linear accelerometry signals (movement) and ambient light (Lux) onto on-board memory.

Actigraphy data will be recorded over two weeks at each measurement point: Baseline Driving Assessment (Weeks 1 and 2), Post-intervention Driving Assessment 2 (Weeks 7 and 8), and during Follow-up Driving Assessment (Weeks 33 and 34).

The following sleep outcomes will be extracted from the actigraphy data;

- Habitual daily sleep duration (minutes of sleep obtained across each 24-h period during data collection)
- Frequency of sleep restriction (frequency count of sleep duration < 6 h s per night each week)

A further extensive list of sleep outcomes are also planned, however, this is the planned initial analysis.

## 3 Analyses

Analyses of change in the primary and secondary outcomes will be conducted on an intention-to-treat (ITT) basis using all randomised participants. The changes in the primary and secondary outcomes will be tested using general linear mixed effects regression models with repeated measures (MMRM; MIXED Procedure in SPSS 22 and STATA 15). These models will include the random intercept for each individual participant and fixed effects for condition (intervention [sleep] vs placebo [nutrition]) and assessment period (baseline, intervention, and 6-month follow-up). Where appropriate, adjustments for demographic variables will be made via their inclusion as additional fixed effects. The outputs of analyses will be reported via significance (as confidence intervals) and magnitude estimates to guide clinical interpretation.

## 4 Discussion

Driving while sleepy represents a critical risk for young drivers. This protocol describes the design of a pragmatic randomised control trial aimed to increase sleep and decrease sleepiness-related road crashes in young adults aged 18–24 years. This study will generate new knowledge about the effectiveness of a sleep extension intervention for reducing risky driving performance in young adults, a cohort at increased risk of sleepiness related road crashes. Secondary analyses, including examination of more complex sleep and circadian components are a critical and planned component of the study and will be explored on a case-by-case basis. This intervention is designed with pragmatic and cost-effective implementation as a major consideration. As such, if effective, this intervention has the potential to be widely and efficiently implemented on a public health level through a range of pathways (e.g., government mandated education about the benefits of sleep for road safety). In turn, widespread policy shift towards prioritising sleep may lead to a reduction in road trauma in young drivers, with subsequent reductions in the medical, industrial, and social costs associated with sleepiness related road-crashes.

### 4.1 Strengths and limitations

There are both strengths and limitations of the proposed study. One strength is the use of standardised and objective measures of sleep and driving in a population of community-dwelling young adults allowing naturalistic, real-time measures of both primary outcomes. Additionally, this is an intensive study which employs a range of measurement techniques to evaluate a range of primary and secondary outcomes including, sleep, risky driving, cognition and risk-taking behaviours. Generalisability of the findings and content of the intervention are specific to self-identifying sleep deprived young adults. However, flexibility of the design should allow it to be successfully adapted for implementation in other settings and groups.

## 5 Conclusion

Young adults are overrepresented in fatigue related road vehicle crashes. However, research and road safety strategies have fallen short in providing answers on how to best tackle this problem. Results from this pragmatic randomised control trial will potentially provide a framework for the successful implementation of a cost-effective and efficient sleep extension protocol to reduce fatigue-related crashes in young adults.

## Funding declaration

This study was funded by a grant from the Australian Government through the National Health and Medical Research Council (APP1163614).

## Ethics declaration

This study was reviewed and approved by the University of Queensland Human Research Ethics Committee, with the approval number: 2019/HE001269. All participants provided informed consent to participate in the study. Furthermore, all participants provided informed consent for the publication of their anonymized case details and images.

## Data availability statement

There is no data presented in this publication. As such, please contact the research team regarding data availability.

## CRediT authorship contribution statement

**Simon S. Smith:** Writing – review & editing, Writing – original draft, Supervision, Resources, Project administration, Methodology, Investigation, Funding acquisition, Formal analysis, Data curation, Conceptualization. **Kalina R. Rossa:** Writing – review & editing, Writing – original draft, Supervision, Resources, Project administration, Methodology, Investigation, Formal analysis, Data curation, Conceptualization. **Shamsi Shekari Soleimanloo:** Writing – review & editing, Writing – original draft, Supervision, Software, Project administration, Methodology, Investigation, Formal analysis, Data curation, Conceptualization. **Cassandra L. Pattinson:** Writing – review & editing, Writing – original draft, Supervision, Resources, Project administration, Methodology, Investigation, Formal analysis, Data curation. **Dwayne L. Mann:** Writing – review & editing, Writing – original draft, Supervision, Software, Project administration, Methodology, Investigation, Formal analysis, Data curation. **Shannon L. Edmed:** Writing – review & editing, Writing – original draft, Project administration, Methodology, Formal analysis, Data curation. **Paul M. Salmon:** Writing – review & editing, Writing – original draft, Supervision, Methodology, Funding acquisition, Data curation, Conceptualization. **Karen A. Sullivan:** Writing – review & editing, Writing – original draft, Supervision, Software, Project administration, Methodology, Funding acquisition, Data curation, Conceptualization.

## Declaration of competing interest

The authors declare that they have no known competing financial interests or personal relationships that could have appeared to influence the work reported in this paper.

## List of abbreviations

AASM: American Academy of Sleep Medicine
ECG: Electrocardiogram
EEG: Electroencephalogram
G-force: Gravitational force
ITT: Intention-to-treat
MMRM: Mixed model repeated measures
NHMRC: National Health and Medical Research Council
NIH Toolbox: National Institute of Health Toolbox
PROMIS: Patient-Reported Outcomes Measurement Information System
