# Supplementary material for: Reducing crash risk for young drivers: Protocol for a pragmatic randomised controlled trial to improve young driver sleep
Source: Heliyon. 2024 Feb 24;10(5):e27066. doi: 10.1016/j.heliyon.2024.e27066 (PMC10920379; doi:10.1016/j.heliyon.2024.e27066)
Supplement: Multimedia component 1 [file mmc1.docx]

| **Construct** | **Questionnaire Name** |
| --- | --- |
| Sleep quality | PROMIS Sleep Disturbance - Short forms 8a & 8b |
| Daytime sleepiness | PROMIS Sleep-related Impairment - Short form 8a |
| Anxiety | PROMIS Anxiety – Short form 8a |
| Depression | PROMIS Depression – Short form 8b |
| Alcohol use | PROMIS Alcohol Use – Short form 7a |
| Social Isolation | PROMIS Social Isolation – Short form 6a |
| Perceived Stress | NIH Toolbox Item bank - Perceived Stress (18+) |
| Bedtime Procrastination | Bedtime Procrastination Scale |
| Emotion Regulation | Emotion Regulation Questionnaire |

Supplementary Table 1. List of secondary outcome (questionnaire) measures collected during laboratory sessions.
